# PNLDC1 catalysis and postnatal germline function are required for piRNA trimming, LINE1 silencing, and spermatogenesis in mice

**DOI:** 10.1371/journal.pgen.1011429

**Published:** 2024-09-23

**Authors:** Chao Wei, Xiaoyuan Yan, Jeffrey M. Mann, Ruirong Geng, Qianyi Wang, Huirong Xie, Elena Y. Demireva, Liangliang Sun, Deqiang Ding, Chen Chen

**Affiliations:** 1 Department of Animal Science, Michigan State University, East Lansing, Michigan, United States of America; 2 Shanghai Key Laboratory of Maternal and Fetal Medicine, Clinical and Translational Research Center, Shanghai First Maternity and Infant Hospital, Frontier Science Center for Stem Cell Research, School of Life Sciences and Technology, Tongji University, Shanghai, China; 3 Department of Chemistry, Michigan State University, East Lansing, Michigan, United States of America; 4 Transgenic and Genome Editing Facility, Institute for Quantitative Health Science & Engineering, Michigan State University, East Lansing, Michigan, United States of America; 5 Reproductive and Developmental Sciences Program, Michigan State University, East Lansing, Michigan, United States of America; 6 Department of Obstetrics, Gynecology and Reproductive Biology, Michigan State University, Grand Rapids, Michigan, United States of America; University of Pennsylvania, UNITED STATES OF AMERICA

## Abstract

PIWI-interacting RNAs (piRNAs) play critical and conserved roles in transposon silencing and gene regulation in the animal germline. Three distinct piRNA populations are present during mouse spermatogenesis: fetal piRNAs in fetal/perinatal testes, pre-pachytene and pachytene piRNAs in postnatal testes. PNLDC1 is required for piRNA 3’ end maturation in multiple species. However, whether PNLDC1 is the bona fide piRNA trimmer and the physiological role of 3’ trimming of different piRNA populations in spermatogenesis in mammals remain unclear. Here, by inactivating *Pnldc1* exonuclease activity *in vitro* and in mice, we reveal that the PNLDC1 trimmer activity is essential for spermatogenesis and male fertility. PNLDC1 catalytic activity is required for both fetal and postnatal piRNA 3’ end trimming. Despite this, postnatal piRNA trimming but not fetal piRNA trimming is critical for LINE1 transposon silencing. Furthermore, conditional inactivation of *Pnldc1* in postnatal germ cells causes LINE1 transposon de-repression and spermatogenic arrest in mice, indicating that germline-specific postnatal piRNA trimming is essential for transposon silencing and germ cell development. Our findings highlight the germ cell-intrinsic role of PNLDC1 and piRNA trimming in mammals to safeguard the germline genome and promote fertility.

## Author summary

PNLDC1 is required for piRNA trimming and is essential for spermatogenesis and male fertility in mice. Loss-of-function mutations in human *PNLDC1* cause azoospermia in men. However, the precise functions of PNLDC1 exonuclease activity in piRNA trimming and germ cell development remain unknown. Here we genetically inactivate the trimmer activity of PNLDC1 in mice and show that the catalytic activity of PNLDC1 is critical for male fertility. Strikingly, postnatal germline conditional deletion of *Pnldc1* in mice that disrupt pre-pachytene and pachytene piRNA trimming causes transposon activation and male infertility. Collectively, these new genetic models provide the proof-of-principle evidence that ablating the catalytic activity of a key piRNA pathway factor in adult males as a novel strategy for non-hormonal male contraception.

## Introduction

PIWI-interacting RNAs (piRNAs) are recognized for their critical and conserved roles in silencing transposons and regulating genes within the germline, which are crucial for maintaining fertility across animal species [1–5]. These small RNAs, bound by PIWI proteins, function as guides in RNA-induced silencing complexes to regulate gametogenesis [6–10]. In mammals, disruptions in the piRNA pathway in germ cells are directly linked to spermatogenic impairments and consequent male infertility [11–16].

During mammalian spermatogenesis, three distinct piRNA populations are expressed at different germ cell developmental stages. In mice, the initial wave, known as fetal piRNAs or prenatal piRNAs, is characterized by a high enrichment of transposon sequences. These piRNAs are associated with MILI and MIWI2 and play a critical role in transposon silencing, thereby safeguarding the integrity of the germline genome [4,17,18]. The second wave of piRNAs, known as pre-pachytene piRNAs, emerges after birth. These piRNAs are associated with MILI and derived mainly from transposons and mRNAs [14]. At the onset of meiosis, specifically during the pachytene stage, a unique and abundant group of piRNAs exclusive to mammals emerges, known as pachytene piRNAs. They are produced from distinct intergenic piRNA clusters and differ significantly from their fetal counterparts in that they are poor in transposon sequences [19–21]. Pachytene piRNAs, in association with MILI and MIWI, are instrumental in regulating the differentiation and maturation of meiotic and post-meiotic germ cells. This regulation is pivotal for the successful completion of spermatogenesis and the production of viable spermatozoa [22–30]. Here we term postnatal pre-pachytene piRNAs and pachytene piRNAs as postnatal piRNAs.

The production of both fetal piRNAs and postnatal piRNAs necessitates a conserved piRNA biogenesis machinery, which efficiently processes long single-stranded piRNA precursors through sequential steps, resulting in the formation of mature piRNAs associated with PIWI proteins [1,3,31,32]. In mammalian male germ cells, piRNA precursor processing predominantly occurs within the intermitochondrial cement (IMC), also known as nuage or germ granules [33–36]. This process involves a sequence of endonucleolytic and exonucleolytic cleavages, resulting in the formation of mature PIWI-loaded piRNAs, which are typically 24–32 nucleotides in length. During this process, after pre-piRNAs are loaded onto PIWI proteins, a crucial trimming step occurs, where the 3’ end of the pre-piRNAs is shortened through 3’-5’ exonuclease activity [37–43]. The final stage of piRNA biogenesis involves the addition of a 2’-O-methyl group to the 3’ end of trimmed piRNAs by methyltransferase HENMT1. This modification confers stability to the piRNAs, thereby preparing them for the protective and regulatory functions within the germline [44–46].

The PARN family of exonucleases plays pivotal roles in piRNA 3’ trimming and maturation in diverse species. In *C*. *elegans*, PARN-1 controls piRNA 3’ length [38]. In silkworms, PARN-like domain containing 1 (PNLDC1) is essential for the 3’ trimming of pre-piRNAs [39]. In mice and humans, despite the presence of both *PARN* and *PNLDC1* in the genome, PNLDC1 is required for piRNA 3’ trimming [40–42,47]. As a member of the PARN family of RNases, PNLDC1 possesses a conserved CAF1 exonuclease domain. In mice, PNLDC1 interacts with TDRKH, and the loss of either PNLDC1 or TDRKH results in piRNA 3’ end extension [39,40,48–50]. PNLDC1 is essential for male fertility in mice, with its loss leading to spermatogenic arrest at the elongated spermatid stage, culminating in azoospermia [40–42]. Recent research has reported various *PNLDC1* variants in azoospermic men, suggesting a monogenic cause of human male infertility [47,51–53]. These mutations lead to an increase in piRNA length due to defective trimming activity of PNLDC1. In the context of piRNA maturation, both fetal piRNAs in prospermatogonia and pre-pachytene piRNAs and pachytene piRNAs in postnatal germ cells undergo crucial 3’ end trimming. The depletion of PNLDC1 leads to elongation in all piRNA populations [40,42]. However, it is unclear how these individual piRNA population defects contribute to the spermatogenic arrest phenotype observed in *Pnldc1* knockout mice. Notably, the loss of PNLDC1 results in the de-repression of LINE1 in adult male germ cells but not in neonatal prospermatogonia [40]. This discrepancy raises further questions whether fetal piRNA 3’ maturation contributes to efficient transposon silencing, thus necessitating deeper investigation into the roles of PNLDC1 in the mammalian piRNA pathway.

In this study, we generated *Pnldc1* catalytic mutant mice to elucidate the physiological role of PNLDC1 exonuclease activity *in vivo*. Our findings demonstrate that PNLDC1 catalysis is required for piRNA trimming, spermatogenesis and male fertility, proving PNLDC1 is indeed the piRNA trimmer in mice. Additionally, conditional deletion of *Pnldc1* in postnatal germ cells in mice disrupts LINE1 transposon silencing and spermatogenesis, highlighting the importance of germline-specific role of PNLDC1 and postnatal piRNA maturation in promoting spermatogenesis.

## Results

### E30A mutation ablates the exonuclease activity of mouse PNLDC1 *in vitro*

**Fig 1:**
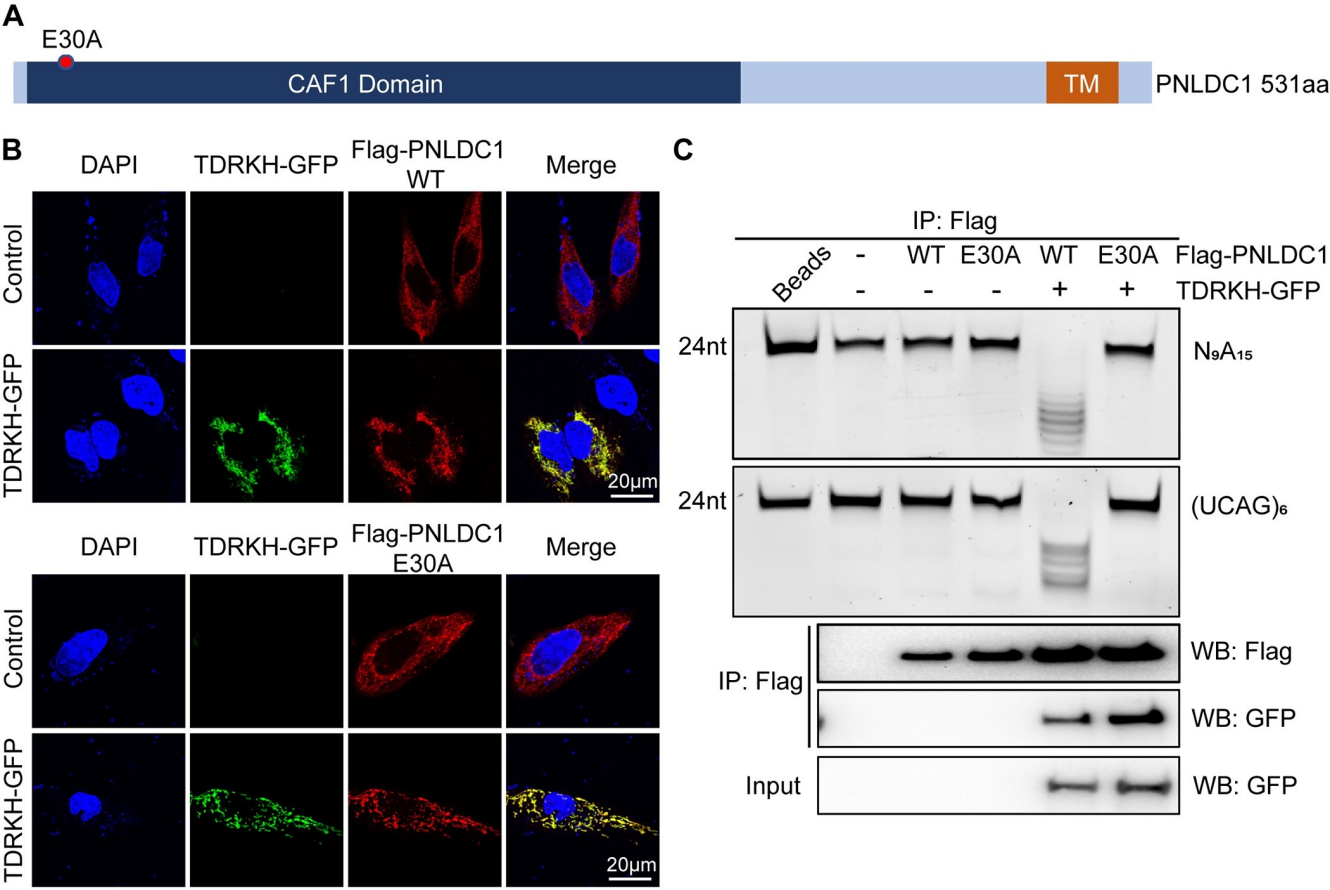
E30A mutation disrupts the exonuclease activity of PNLDC1 *in vitro*. **(A)** A schematic diagram of PNLDC1 protein domains and E30A mutation. **(B)** TDRKH recruits PNLDC1 and PNLDC1 E30A to mitochondria. HeLa cells were transfected with Flag-tagged PNLDC1/PNLDC1 E30A alone or together with TDRKH-GFP plasmids. After 24 h, the cells were fixed and stained with anti-Flag antibody. DNA was stained with DAPI. Scale bar, 20 μm. **(C)** 293T cells were transfected with plasmids expressing wild-type or catalytically inactive (E30A) PNLDC1, and the cell lysates were immunoprecipitated and analyzed by Western blotting (bottom). *In vitro* trimming assay was performed to detect the exonuclease activity of wild-type or catalytically inactive (E30A) PNLDC1. Results shown in (B) and (C) are representative of 3 biological replicates.

PNLDC1 contains a conserved CAF1 exonuclease domain with a key DEDD (Asp-Glu-Asp-Asp) motif required for catalytic activity (Fig 1A). Mutation of the DEDD motif causes PNLDC1 exonuclease inactivation in silkworm [39]. To test whether mouse PNLDC1 exhibits exonuclease activity *in vitro* and whether it is catalyzed by the CAF1 nuclease domain, we mutated the 30^th^ Glu to Ala in the DEDD motif of mouse PNLDC1 (PNLDC1 E30A) (Fig 1A) [39]. We first examined the expression and localization of ectopically expressed wildtype PNLDC1 and PNLDC1 E30A in HeLa cells. When singly expressed, Flag-tagged PNLDC1 (Flag-PNLDC1) and Flag-PNLDC1 E30A exhibited similar expression level and subcellular localization pattern (Fig 1B). When co-expressed with GFP-tagged TDRKH (TDRKH-GFP), mitochondrial-anchored TDRKH recruited both PNLDC1 and PNLDC1 E30A to the mitochondria (Fig 1B). This indicates that the E30A mutation does not affect mutant PNLDC1 protein expression, stability, and its recruitment to mitochondria by TDRKH to participate in piRNA trimming. To test the trimmer activity of PNLDC1, we next established a robust *in vitro* PNLDC1 trimming assay by combining the 293T cell-based heterologous expression system with an *in vitro* RNA trimming assay. In this system, Flag-PNLDC1 and TDRKH-GFP were co-expressed in 293T cells and PNLDC1-TDRKH complexes were immunoprecipitated by Flag-antibody conjugated beads to incubate with synthesized RNA substrates to detect RNA trimming. We showed that PNLDC1 alone was unable to trim RNA substrates but displayed strong trimming activity in the presence of TDRKH (Fig 1C). This is consistent with previously reported PNLDC1 activity in the silkworm [39]. Strikingly, PNLDC1 E30A mutation completely disrupted the PNLDC1 trimming activity without affecting its interaction with TDRKH (Fig 1C). We confirmed this finding using two independent RNA oligonucleotides as substrates (Fig 1C). Together, these data demonstrate that mouse PNLDC1 possesses trimmer activity and the E30A mutation abolishes its exonuclease activity *in vitro*.

### PNLDC1 exonuclease inactivation causes LINE1 upregulation and male infertility in mice

**Fig 2:**
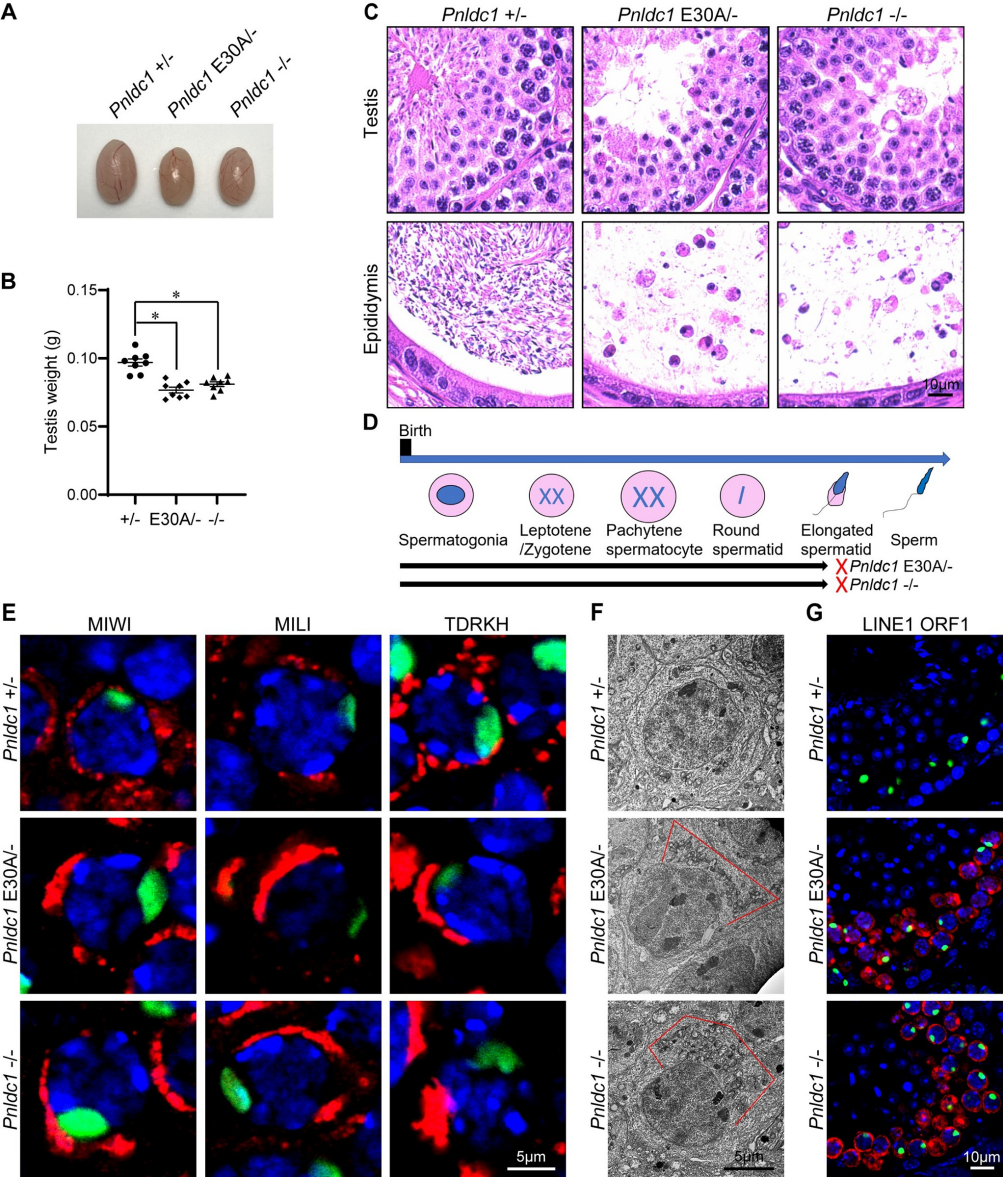
*Pnldc1* E30A mutation causes spermatocyte mitochondrial aggregation, LINE1 derepression, and spermatogenic arrest in mice. **(A)** Images of adult *Pnldc1*^*+/-*^, *Pnldc1*^*E30A/-*^, and *Pnldc1*^*-/-*^ testes. **(B)** Testis weights of adult *Pnldc1*^*+/-*^, *Pnldc1*^*E30A/-*^, and *Pnldc1*^*-/-*^ mice are shown. n = 8. Error bars represent SEM. The P-value was calculated by unpaired t-test. *, P < 0.01. **(C)** Hematoxylin and eosin-stained testis and epididymis sections from adult *Pnldc1*^*+/-*^, *Pnldc1*^*E30A/-*^, and *Pnldc1*^*-/-*^ mice are shown. Scale bar, 10μm. **(D)** Spermatogenic arrest stage in *Pnldc1*^*E30A/-*^ and *Pnldc1*^*-/-*^ mice. **(E)** Co-immunostaining of MIWI, MILI, or TDRKH (red) with γH2AX (green) in adult *Pnldc1*^*+/-*^, *Pnldc1*^*E30A/-*^, and *Pnldc1*^*-/-*^ spermatocytes. DNA was stained with DAPI. Scale bar, 5μm. **(F)** Transmission electron microscopy was performed on pachytene spermatocytes from adult *Pnldc1*^*+/-*^, *Pnldc1*^*E30A/-*^, and *Pnldc1*^*-/-*^ testes. The mitochondria aggregation is indicated by red line. Scale bar, 5μm. **(G)** Co-immunostaining of LINE1 ORF1 (red) with γH2AX (green) in adult *Pnldc1*^*+/-*^, *Pnldc1*^*E30A/-*^, and *Pnldc1*^*-/-*^ spermatocytes. DNA was stained with DAPI. Scale bar, 10μm. Results shown in (C) and (E)-(G) are representative of 3 biological replicates.

To investigate whether PNLDC1 trimmer activity is essential for piRNA trimming and spermatogenesis *in vivo*, we generated a *Pnldc1* E30A mutant allele (*Pnldc1*^*E30A*^) in mice using CRISPR-Cas9 genome editing (S1A Fig). The mutation in *Pnldc1*^*E30A*^ mice was confirmed by Sanger DNA sequencing (S1B Fig). We further generated *Pnldc1*^*E30A/-*^ mice by breeding the *Pnldc1*^*E30A*^ allele into the *Pnldc1* null (*Pnldc1*^*-*^) background [40]. *Pnldc1*^*E30A/-*^ mice were viable and developed normally but exhibited mildly reduced testis mass compared to *Pnldc1*^*+/-*^ control littermates (Fig 2A and 2B). This reduction in testis weight is similar to that observed in *Pnldc1*^*-/-*^ testes [40]. Histological analysis of spermatogenesis revealed a spermatogenic arrest at the elongated spermatid stage in *Pnldc1*^*E30A/-*^ testes, a spermatogenic failure that phenocopies *Pnldc1*^*-/-*^ mice (Fig 2C and 2D). As a result, in *Pnldc1*^*E30A/-*^ epididymis, only sloughed spermatids and residual cytoplasm were observed without normal spermatozoa being present, leading to male infertility (Fig 2C).

In adult testes, piRNA precursors are processed to load onto MIWI and MILI in the IMC among mitochondrial clusters in pachytene spermatocytes. The piRNA biogenesis machinery comprises multiple factors including the PNLDC1/TDRKH trimming complex. To confirm whether PNLDC1-TDRKH interaction is affected by the E30A mutation in germ cells, we performed TDRKH immunoprecipitation and mass spectrometry (IP-MS). MS results showed that PNLDC1 were abundantly detected in TDRKH immunoprecipitation in both *Pnldc1*^*+/-*^ and *Pnldc1*^*E30A/-*^ testes (S1, S2 and S3 Tables). These data indicate that PNLDC1 E30A mutation does not affect its expression and interaction with TDRKH *in vivo*. We next examined the expression and localization of MIWI, MILI, and TDRKH in *Pnldc1*^*E30A/-*^ testes. Western blotting showed similar expression levels of TDRKH and MILI in *Pnldc1*^*+/-*^, *Pnldc1*^*E30A/-*^, and *Pnldc1*^*-/-*^ testes (S2A Fig). However, MIWI protein level was decreased in *Pnldc1*^*E30A/-*^ testes, representing a similar MIWI reduction observed in *Pnldc1*^*-/-*^ testes (S2A Fig). Immunofluorescence showed that MIWI, MILI, and TDRKH localized to polarized and aggregated large perinuclear granules in *Pnldc1*^*E30A/-*^ pachytene spermatocytes, suggesting mitochondrial aggregation in these cells (Figs 2E and S2B). Transmission electron microscopy confirmed our observation: mitochondria in *Pnldc1*^*E30A/-*^ spermatocytes displayed clustered aggregation and polar distribution, a defect also observed in *Pnldc1*^*-/-*^ spermatocytes (Fig 2F). Transmission electron microscopy results showed that chromatoid bodies in *Pnldc1*^*E30A/-*^ round spermatids were slightly reduced in size and electron density but with no obvious fragmentation, which is similar to *Pnldc1*^*-/-*^ (S2C Fig). Since PNLDC1 is required for LINE1 transposon silencing [40, 41], we next tested whether the trimmer activity of PNLDC1 is crucial for this role. By immunostaining, transposon LINE1 ORF1 protein was significantly upregulated in *Pnldc1*^*E30A/-*^, indicating that the trimmer activity of PNLDC1 is indispensable for LINE1 suppression (Fig 2G). Taken together, we conclude that PNLDC1 trimmer activity is required for LINE1 transposon silencing, spermatogenesis, and male fertility in mice.

### PNLDC1 exonuclease activity is essential for postnatal piRNA trimming

**Fig 3:**
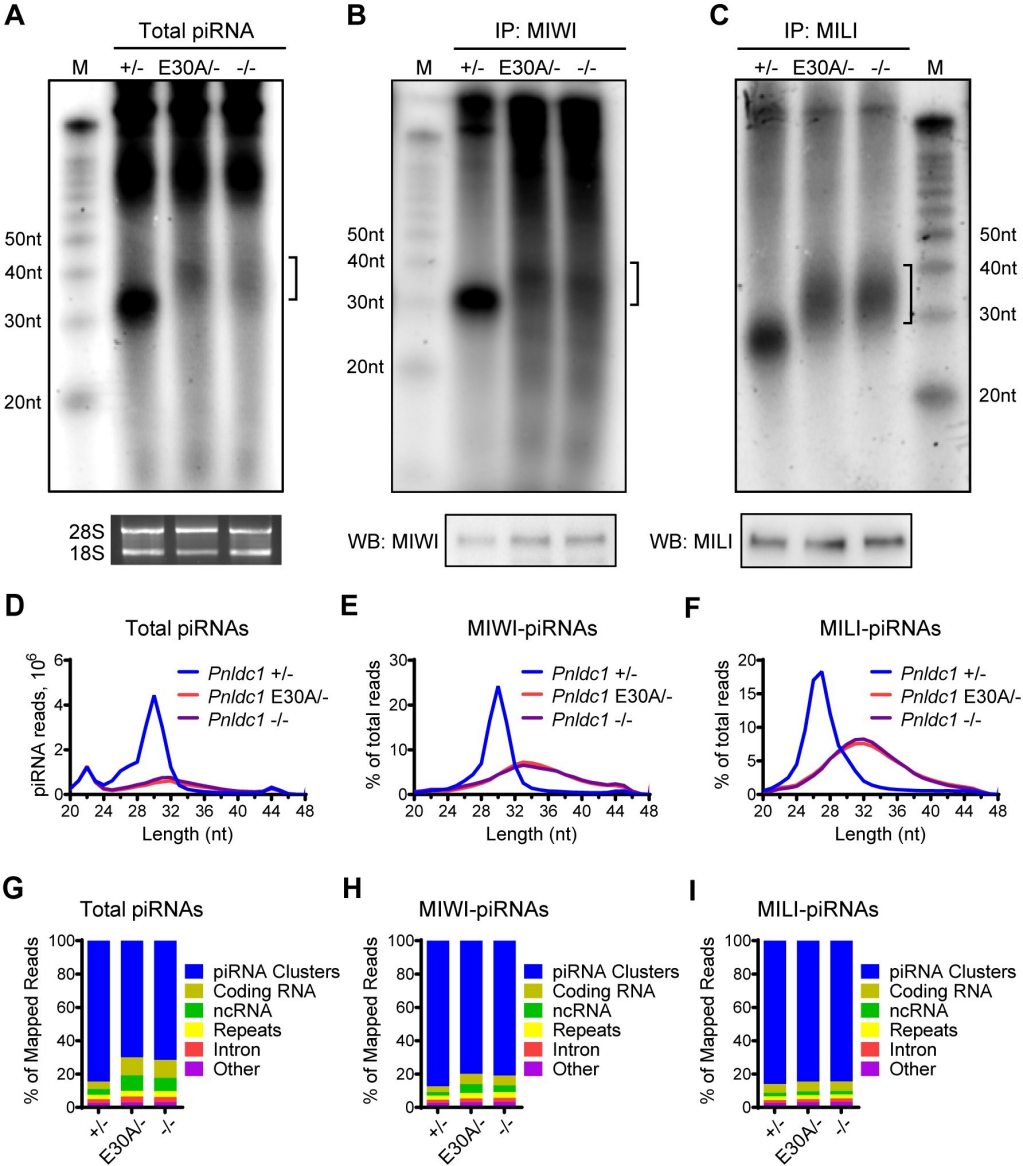
*Pnldc1* E30A mutation triggers defective pre-piRNA trimming. **(A)** Total RNAs extracted from adult *Pnldc1*^*+/-*^, *Pnldc1*^*E30A/-*^, and *Pnldc1*^*-/-*^ testes were end-labeled with [^32^P]-ATP, separated by 15% TBE urea gel, and detected by autoradiography. Square bracket indicates extended piRNAs. The 18S and 28S ribosomal RNAs served as loading controls. **(B)** RNAs were isolated from immunoprecipitated MIWI RNPs from adult testes, end-labeled with [^32^P]-ATP, separated by 15% TBE urea gel, and detected by autoradiography. Western blotting was performed with anti-MIWI antibody to show immunoprecipitation efficiency. Square brackets indicate extended piRNAs. **(C)** RNAs were isolated from immunoprecipitated MILI RNPs from adult testes, end-labeled with [^32^P]-ATP, separated by 15% TBE urea gel, and detected by autoradiography. Western blotting was performed with anti-MILI antibody to show immunoprecipitation efficiency. Square bracket indicates extended piRNAs. **(D)** The length distribution of small RNAs from adult *Pnldc1*^*+/-*^, *Pnldc1*^*E30A/-*^, and *Pnldc1*^*-/-*^ testicular total small RNA libraries. Data were normalized by microRNA reads (21–23 nt). **(E)** The length distribution of MIWI-bound piRNAs from adult *Pnldc1*^*+/-*^, *Pnldc1*^*E30A/-*^, and *Pnldc1*^*-/-*^ MIWI-piRNA libraries. **(F)** The length distribution of MILI-bound piRNAs from adult *Pnldc1*^*+/-*^, *Pnldc1*^*E30A/-*^, and *Pnldc1*^*-/-*^ MILI-piRNA libraries. **(G)** Genomic annotation of total piRNAs from adult *Pnldc1*^*+/-*^, *Pnldc1*^*E30A/-*^, and *Pnldc1*^*-/-*^ testes. Sequence reads (24–48 nt) from total piRNA libraries were aligned to mouse genomic sequence sets in the following order: piRNA clusters, coding RNA, non-coding RNA, repeats, intron, and other. **(H)** Genomic annotation of MIWI-bound piRNAs from adult *Pnldc1*^*+/-*^, *Pnldc1*^*E30A/-*^, and *Pnldc1*^*-/-*^ testes. Sequence reads (24–48 nt) from MIWI-piRNA libraries were aligned to mouse genomic sequence sets in the following order: piRNA clusters, coding RNA, non-coding RNA, repeats, intron, and other. **(I)** Genomic annotation of MILI-bound piRNAs from adult *Pnldc1*^*+/-*^, *Pnldc1*^*E30A/-*^, and *Pnldc1*^*-/-*^ testes. Sequence reads (24–48 nt) from MILI-piRNA libraries were aligned to mouse genomic sequence sets in the following order: piRNA clusters, coding RNA, non-coding RNA, repeats, intron, and other. Results shown in (A)-(C) are representative of 3 biological replicates.

We next investigated the effect of PNLDC1 exonuclease inactivation on postnatal piRNA biogenesis. We used RNA labeling to examine the abundance and size of piRNA populations from adult *Pnldc1*^*+/-*^, *Pnldc1*^*E30A/-*^, and *Pnldc1*^*-/-*^ testes. Radiolabeling of total RNA showed a normal piRNA population of around 30 nt in length in *Pnldc1*^*+/-*^ testes (Fig 3A). However, an abnormally longer but low abundant small RNA population of 30–40 nt in length was observed *Pnldc1*^*E30A/-*^ testes, consistent with the extended piRNA population in *Pnldc1*^*-/-*^ testes (Fig 3A). We further performed MIWI and MILI immunoprecipitation and isolated MIWI-bound piRNAs (MIWI-piRNAs) and MILI-bound piRNAs (MILI-piRNAs) from *Pnldc1*^*+/-*^, *Pnldc1*^*E30A/-*^, and *Pnldc1*^*-/-*^ testes. Radiolabeling assay showed that MIWI-piRNAs and MILI-piRNAs from *Pnldc1*^*E30A/-*^ testes were similar to those from *Pnldc1*^*-/-*^, and both were longer than corresponding piRNAs in *Pnldc1*^*+/-*^ (Fig 3B and 3C). We further sequenced small RNA libraries constructed from total piRNA, MIWI-piRNAs and MILI-piRNAs in these mice and observed the same trend: a significant decrease in total piRNA amount (Fig 3D) and an upshift in piRNA lengths in *Pnldc1*^*E30A/-*^ and *Pnldc1*^*-/-*^ compared to *Pnldc1*^*+/-*^ controls (Fig 3D, 3E and 3F). Importantly, at the piRNA population level, piRNA defects of *Pnldc1*^*E30A/-*^ and *Pnldc1*^*-/-*^ were indistinguishable.

We further characterized the extended piRNAs in *Pnldc1*^*E30A/-*^ testes by mapping the reads to the mouse genome. *Pnldc1*^*E30A/-*^ piRNAs resembled *Pnldc1*^*-/-*^ piRNAs in several features: A) mainly mapped to piRNA clusters (Fig 3G, 3H and 3I); B) strong U bias at the first nucleotide position, which is the 5’ end signature of wild-type pachytene piRNAs (S3A Fig); C) piRNA 3’ end extension (S3B Fig). Collectively, these data demonstrate that the PNLDC1 exonuclease activity is required for postnatal pre-piRNA 3’ end trimming and therefore PNLDC1 is indeed the piRNA trimmer in mice.

### PNLDC1 exonuclease activity is required for fetal piRNA trimming

**Fig 4:**
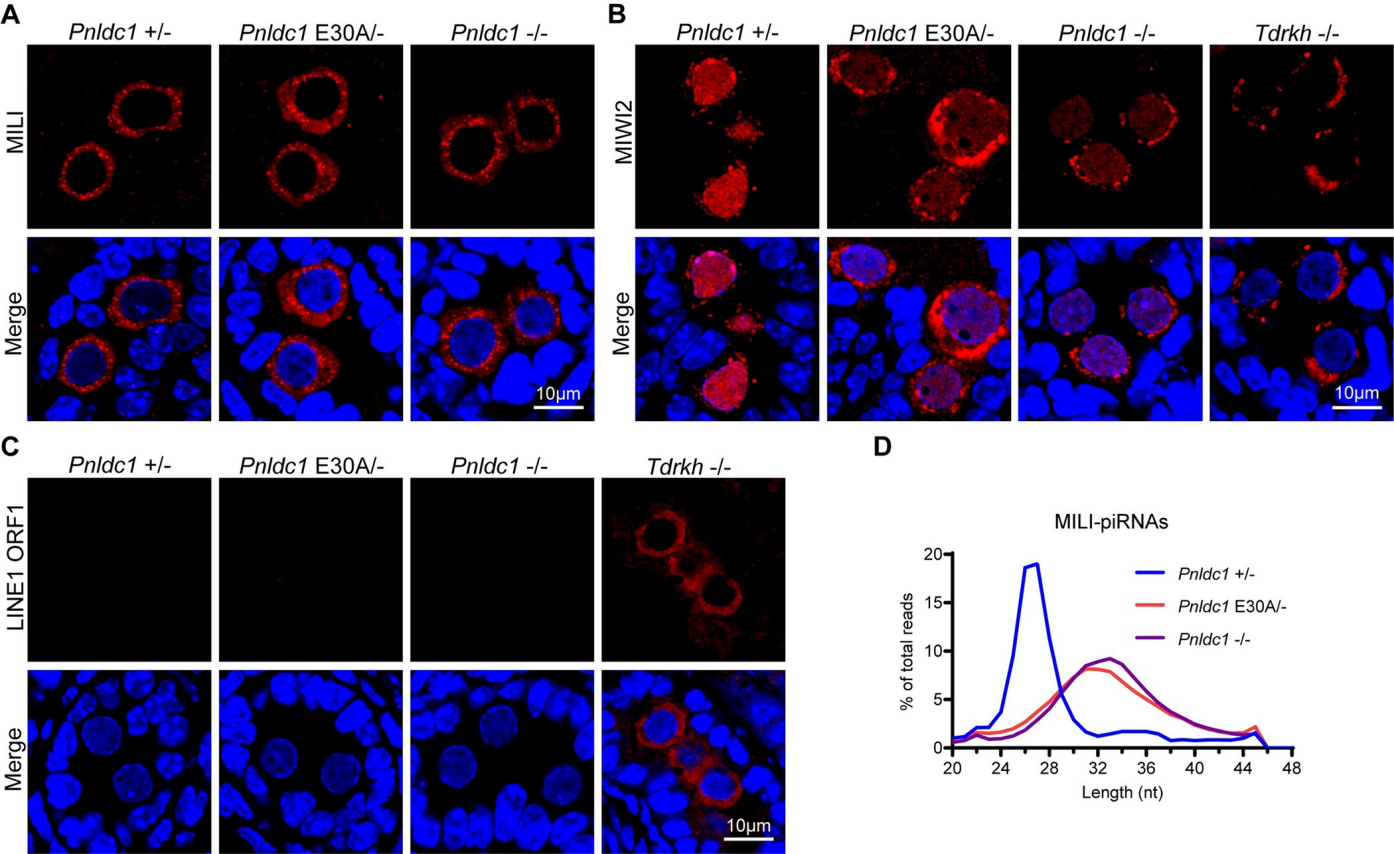
*Pnldc1* E30A mutation causes MIWI2 mislocalization but not LINE1 activation in neonatal prospermatogonia. **(A)** Immunostaining of MILI on neonatal (P0) testis sections from *Pnldc1*^*+/-*^, *Pnldc1*^*E30A/-*^, and *Pnldc1*^*-/-*^ mice. DNA was stained with DAPI. Scale bar, 10μm. **(B)** Immunostaining of MIWI2 on neonatal (P0) testis sections from *Pnldc1*^*+/-*^, *Pnldc1*^*E30A/-*^, *Pnldc1*^*-/-*^, and *Tdrkh*^*-/-*^ mice. DNA was stained with DAPI. Scale bar, 10μm. **(C)** Immunostaining of LINE1 ORF1 on neonatal (P0) testis sections from *Pnldc1*^*+/-*^, *Pnldc1*^*E30A/-*^, *Pnldc1*^*-/-*^, and *Tdrkh*^*-/-*^ mice. DNA was stained with DAPI. Scale bar, 10μm. **(D)** The length distribution of MILI-bound piRNAs from neonatal (P0) *Pnldc1*^*+/-*^, *Pnldc1*^*E30A/-*^, and *Pnldc1*^*-/-*^ MILI-piRNA libraries. Results shown in (A)-(C) are representative of 3 biological replicates.

Next, we investigated the effect of PNLDC1 exonuclease inactivation on fetal piRNA biogenesis in prospermatogonia. By examining MILI and MIWI2 localization in neonatal (P0) *Pnldc1*^*+/-*^, *Pnldc1*^*E30A/-*^, and *Pnldc1*^*-/-*^ testes, we found that MILI was expressed in a cytoplasmic granular pattern in prospermatogonia of all three genotypes (Fig 4A). While MIWI2 was predominantly localized in the nuclei of *Pnldc1*^*+/-*^ prospermatogonia, it was only partially localized to nuclei with majority of signals in the cytoplasm of *Pnldc1*^*E30A/-*^ and *Pnldc1*^*-/-*^ prospermatogonia (Fig 4B). By contrast, MIWI2 was completely cytoplasmic in *Tdrkh*^*-/-*^ prospermatogonia (Fig 4B). The mis-localization of MIWI2 in *Pnldc1*^*E30A/-*^ suggests defects in fetal piRNA biogenesis and function. Because fetal piRNAs are essential for LINE1 silencing, we examined transposon LINE1 expression in *Pnldc1*^*E30A/-*^ prospermatogonia. Similar to *Pnldc1*^*+/-*^ and *Pnldc1*^*-/-*^, no obvious LINE1 ORF1 was observed in *Pnldc1*^*E30A/-*^ prospermatognia (Fig 4C). This contrasts with the significant upregulation of LINE1 in *Tdrkh*^*-/-*^ prospermatogonia and suggests that the remaining nuclear MIWI2 in *Pnldc1*^*E30A/-*^ prospermatogonia is still functional for LINE1 silencing. We further explored whether PNLDC1 exonuclease inactivation affects fetal piRNA trimming by small RNA sequencing of MILI-piRNAs in neonatal (P0) testes. Similarly with results from adult testes, the peak of MILI-piRNAs shifted from 27 nt in *Pnldc1*^*+/-*^ to 31–33 nt in *Pnldc1*^*E30A/-*^ and *Pnldc1*^*-/-*^ testes (Fig 4D). Together, we conclude that PNLDC1 exonuclease activity is required for fetal piRNA trimming, but dispensable for LINE1 silencing in prospermatogonia.

### Postnatal germ cell-specific conditional deletion of *Pnldc1* in mice leads to LINE1 de-repression and spermatogenic arrest

**Fig 5:**
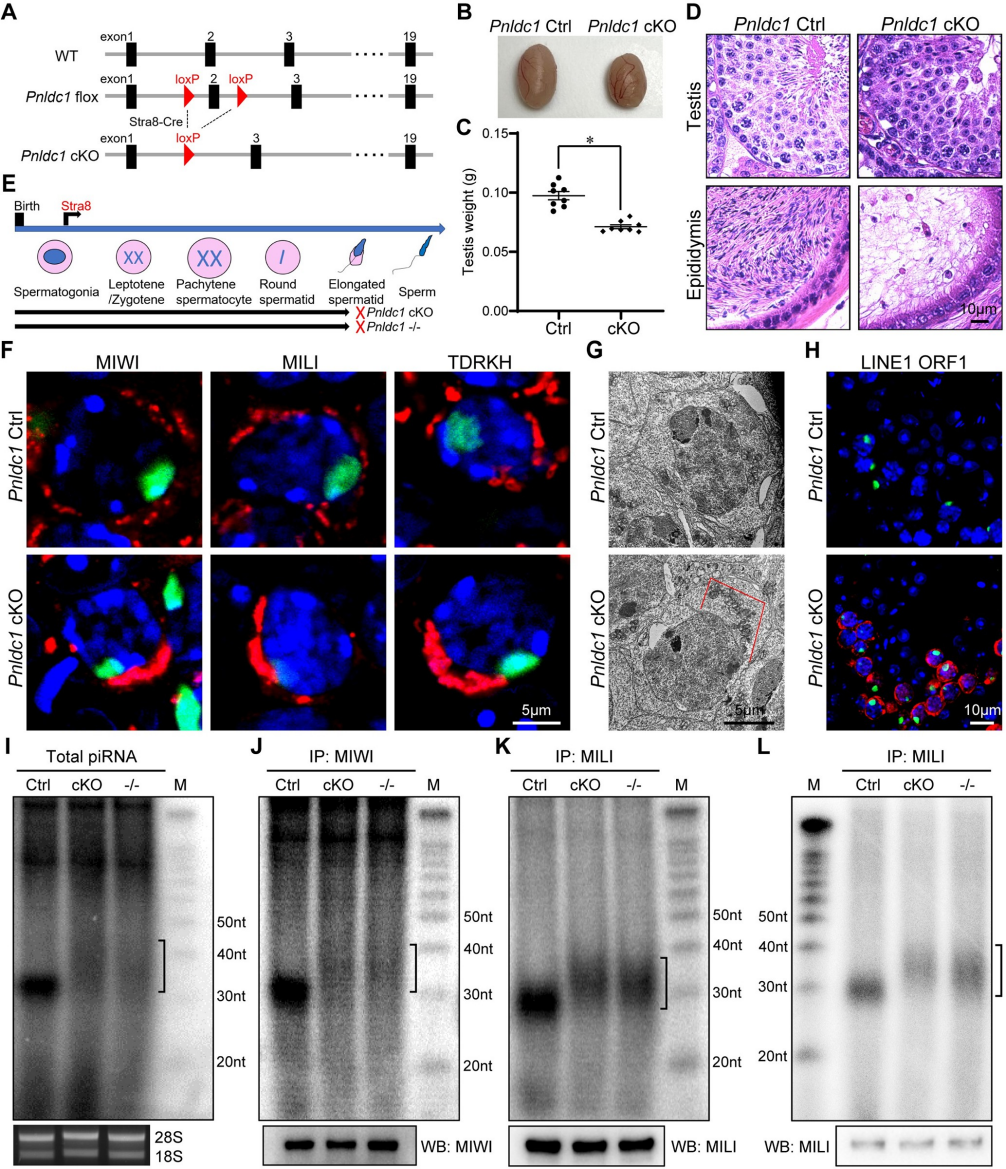
Conditional deletion of *Pnldc1* in postnatal germ cells causes defective piRNA trimming, LINE1 upregulation, and male infertility. **(A)** A schematic diagram showing the gene targeting strategy for the generation of *Pnldc1* cKO. Cre-mediated deletion removed the exon 2 of *Pnldc1*. **(B)** Images of adult control and *Pnldc1* cKO testes. **(C)** Testis weights of adult control and *Pnldc1* cKO mice are shown. n = 8. Error bars represent SEM. The P-value was calculated by unpaired t-test. *, P < 0.01. **(D)** Hematoxylin and eosin-stained testis and epididymis sections from adult control and *Pnldc1* cKO mice are shown. Scale bar, 10μm. **(E)** Spermatogenic arrest stage in *Pnldc1* cKO and *Pnldc1* KO mice. **(F)** Co-immunostaining of MIWI, MILI, or TDRKH (red) with γH2AX (green) in adult control and *Pnldc1* cKO spermatocytes. DNA was stained with DAPI. Scale bar, 5μm. **(G)** Transmission electron microscopy was performed on pachytene spermatocytes from adult control and *Pnldc1* cKO testes. The mitochondria aggregation is indicated by red line. Scale bar, 5μm. **(H)** Co-immunostaining of LINE1 ORF1 (red) with γH2AX (green) in adult control and *Pnldc1* cKO spermatocytes. DNA was stained with DAPI. Scale bar, 10μm. **(I)** Total RNAs extracted from adult control and *Pnldc1* cKO testes were end-labeled with [^32^P]-ATP, separated by 15% TBE urea gel, and detected by autoradiography. Square bracket indicates extended piRNAs. The 18S and 28S ribosomal RNAs served as loading controls. **(J)** RNAs were isolated from immunoprecipitated MIWI RNPs from adult testes, end-labeled with [^32^P]-ATP, separated by 15% TBE urea gel, and detected by autoradiography. Western blotting was performed with anti-MIWI antibody to show immunoprecipitation efficiency. Square bracket indicates extended piRNAs. **(K)** RNAs were isolated from immunoprecipitated MILI RNPs from adult testes, end-labeled with [^32^P]-ATP, separated by 15% TBE urea gel, and detected by autoradiography. Western blotting was performed with anti-MILI antibody to show immunoprecipitation efficiency. Square bracket indicates extended piRNAs. **(L)** RNAs were isolated from immunoprecipitated MILI RNPs from P10 testes, end-labeled with [^32^P]-ATP, separated by 15% TBE urea gel, and detected by autoradiography. Western blotting was performed with anti-MILI antibody to show immunoprecipitation efficiency. Square brackets indicate extended piRNAs. Results shown in (D) and (F)-(L) are representative of 3 biological replicates.

It is unclear whether the spermatogenic arrest and LINE1 transposon de-repression in *Pnldc1*^*E30A/-*^ and *Pnldc1*^*-/-*^ testes is due to the defect of trimming in fetal piRNAs or postnatal piRNAs. To answer this, we generated a *Pnldc1* flox allele in mice using CRISPR-Cas9 genome editing (S4A Fig). The exon 2 of mouse *Pnldc1* was flanked by two loxP sites allowing its deletion by the Cre recombinase to ablate gene function. By combining *Pnldc1* flox with *Stra8*-Cre, we obtained *Stra8*-Cre^+^, *Pnldc1* flox/- conditional knockout (*Pnldc1* cKO) mice in which *Pnldc1* was deleted in male germ cells starting at postnatal day 3 (Fig 5A). This deletion would not affect PNLDC1 expression during fetal piRNA biogenesis but only ablate PNLDC1 in postnatal germ cells. *Pnldc1* cKO mice were viable but exhibited reduced testis mass compared with control mice (Fig 5B and 5C). Histological examination of *Pnldc1* cKO testes revealed that germ cells were primarily arrested at the elongated spermatid stage without normal spermatozoa in the epididymis, phenocopying *Pnldc1*^*-/-*^ germ cell defect (Fig 5D and 5E). These results indicate that the postnatal function of PNLDC1 in germ cells is essential for spermatogenesis and male fertility.

We further examined the expression and localization of MIWI, MILI, and TDRKH in *Pnldc1* cKO testes. Western blotting showed similar expression levels of TDRKH and MILI in control and *Pnldc1* cKO testes (S5A Fig). However, MIWI protein level was decreased in *Pnldc1* cKO testes (S5A Fig). Immunofluorescence showed that MIWI, MILI, and TDRKH formed polarized and aggregated large perinuclear granules in *Pnldc1* cKO pachytene spermatocytes (Figs 5F and S5B). This coincides with observed mitochondrial aggregation in *Pnldc1* cKO spermatocytes by transmission electron microscopy (Fig 5G). Chromatoid bodies in *Pnldc1* cKO round spermatids displayed slight reduction in size and electron density but with no obvious disintegration (S5C Fig). Interestingly, LINE1 ORF1 protein was significantly upregulated in *Pnldc1* cKO spermatocytes as in *Pnldc1*^*-/-*^ mice (Fig 5H). This indicates that postnatally expressed PNLDC1 in germ cells is essential for LINE1 silencing.

We next examined the effect of postnatal deletion of *Pnldc1* in germ cells on piRNA biogenesis. Small RNA labeling revealed that total piRNA from adult *Pnldc1* cKO testes was longer than that from control testes (Fig 5I). Labeling of MIWI-piRNAs and MILI-piRNAs confirmed the presence of longer postnatal piRNA populations in *Pnldc1* cKO testes, resembling the piRNA trimming defect in *Pnldc1*^*-/-*^ testes (Fig 5J and 5K). To test if postnatal germline deletion of *Pnldc1* affects pre-pachytene piRNA trimming, we performed radiolabeling of MILI-piRNAs from postnatal day 10 (P10) *Pnldc1* cKO testes. We found that MILI-bound pre-pachytene piRNAs from P10 *Pnldc1* cKO testes were longer than those from control testes (Fig 5L). The piRNA trimming defect or decreased MIWI level could account for the observed LINE1 derepression in *Pnldc1* cKO testes. To clarify this, we stained LINE1 ORF1 in P18 testes when MIWI is still expressed at a considerable level. LINE1 ORF1 protein was significantly upregulated in P18 *Pnldc1* cKO spermatocytes, similar to its pattern in P18 *Pnldc1*^*-/-*^ and *Pnldc1*^*E30A/-*^ spermatocytes (S5D Fig). Together, these results suggest that proper postnatal piRNA trimming by PNLDC1 is required for LINE1 suppression and spermatogenesis.

## Discussion

Pre-piRNA 3’ trimming catalyzed by the piRNA trimmer is a critical step during piRNA maturation in diverse species. Here, by inactivating the CAF1 nuclease domain of PNLDC1 *in vitro* and in mice we demonstrate that PNLDC1 is a bona fide mammalian pre-piRNA trimmer and this trimmer activity is essential for male fertility. Furthermore, postnatal deletion of *Pnldc1* in male germ cells disrupts spermatogenesis, highlighting the potential of blocking piRNA trimmer activity in adult males for fertility regulation.

*Pnldc1*^*E30A/-*^ and *Pnldc1*^*-/-*^ mice exhibited identical piRNA and spermatogenic defects in all aspects we examined, which include: untrimmed fetal piRNAs and postnatal piRNAs at 3’ ends; normal LINE silencing in neonatal prospermatogonia but LINE1 de-repression in juvenile and adult spermatocytes; adult spermiogenic arrest at the elongated spermatid stage. These results demonstrate that the physiological role of PNLDC1 is tightly coupled with its exonuclease activity in piRNA regulation and spermatogenesis.

Both PNLDC1 and TDRKH are required for piRNA trimming in mice [40–42, 49, 50]. Notably, *Tdrkh*^*-/-*^ mice show more severe defects in piRNA production and spermatogenesis than *Pnldc1*^*-/-*^ mice, indicating that TDRKH has broader functions in piRNA regulation beyond piRNA 3’ end trimming [49]. Our study demonstrates that the PNLDC1 exonuclease domain is crucial for piRNA trimming *in vitro* and *in vivo*, proving PNLDC1 as a bona fide piRNA trimmer. However, *in vitro* trimming assay reveals that PNLDC1 cannot function alone and its trimmer activity depends on the presence of TDRKH. TDRKH is a mitochondrial transmembrane protein capable of recruiting PNLDC1 to mitochondria in cultured cells and we previously showed that it directly binds and recruits MIWI to the IMC for piRNA production [50, 54–56]. Therefore, we propose that TDRKH acts as a mitochondrial scaffold protein to simultaneously engage PNLDC1 and PIWI proteins to regulate piRNA trimming and PIWI function. This model can explain the phenotypic differences between *Tdrkh*^*-/-*^ and *Pnldc1*^*-/-*^ mice. Our findings clarify that PNLDC1 is the piRNA trimming enzyme while TDRKH is not. However, how PNLDC1 and TDRKH interact to form the piRNA trimming complex remains unknown and requires further biochemical and structural investigation.

We show that PNLDC1 catalysis is required for the 3’ trimming of both fetal piRNAs and postnatal piRNAs, distinct piRNA populations from different germ cell developmental stages. However, defective fetal piRNA trimming and postnatal piRNA trimming have different effects on germ cell function. Without PNLDC1 catalytic activity, LINE1 silencing is normal in neonatal prospermatogonia but becomes defective in adult spermatocytes. This suggests that fetal piRNA trimming mediated by PNLDC1 is not required for LINE1 suppression in prospermatogonia. Consistent with this, we observed partial nuclear localization of MIWI2, suggesting that the residual MIWI2-piRNAs in PNLDC1 catalysis-deficient fetal germ cells are still functional in transcriptionally silencing LINE1. Given that gene knockouts of most piRNA biogenesis factors affecting fetal piRNAs cause LINE1 mis-regulation in prospermatogonia and result in early meiotic germ cell arrest [12,13,31,49,57,58], the milder spermatogenic defect in *Pnldc1*^*E30A/-*^ and *Pnldc1*^*-/-*^ mice could result from defective postnatal piRNA maturation alone.

One important advance is the clarification of germ cell-specific function of PNLDC1 by conditional deletion of *Pnldc1* in postnatal germ cells. *Pnldc1*^*cKO*^ mice exhibit identical LINE1 de-repression and spermatogenic arrest as *Pnldc1*^*-/-*^ mice. This not only excludes its potential other functions beyond the germline on spermatogenesis but also reveals the requirement of postnatal piRNA trimming in male fertility. Recently multiple PNLDC1 variants with defective piRNA processing have been detected in azoospermia in men, establishing a PNLDC1-specific monogenic cause of human infertility [47,51–53]. Thus, based on our genetic dissection of mammalian PNLDC1 catalysis and its germ cell specific function, we propose targeted inhibition of PNLDC1 enzymatic activity as a novel means for non-hormonal male contraception.

## Materials and methods

### Ethics statement

All animal procedures were approved by the Institutional Animal Care and Use Committee of Michigan State University. All experiments with mice were conducted ethically according to the Guide for the Care and Use of Laboratory Animals and institutional guidelines.

### Mouse strains

*Pnldc1*^*E30A*^ mutant and *Pnldc1* flox mice were generated by Michigan State University’s Transgenic and Genome Editing Facility. *Pnldc1*^*E30A*^ mutant mice were generated by CRISPR-Cas9 targeting of the mouse *Pnldc1* locus (ENSMUSG00000073460). Wild-type NLS-Cas9 protein, synthetic crRNA, tracrRNA, and single-stranded oligodeoxynucleotide (ssODN) donor template from Integrated DNA Technologies (Coralville, IA, USA) were used. Protospacer (N)_20_ and protospacer adjacent motif (PAM) sequences corresponding to the crRNA used were 5’-GGTCTGGATATAGAGTTCAC*-*AGG-3’. Donor ssODN in the reverse orientation had the following sequence: 5’- GGTAAGACAGTAATTAGTACCTGATCTGTTGGGGCCGAGACAAGTTTGAACGCAGACCTGTGAAAGCTATATCCAGACCTACGGGAGCACAAAACAGAC-3’. Synthetic crRNA and tracrRNA were incubated at 95°C for 5 min and cooled down to form RNA heteroduplexes, which were then incubated with Cas9 protein for 5 min at 37°C to preform ribonucleoprotein (RNP) complexes. RNPs along with the ssODN were electroporated into C57BL/6 mouse zygotes using a Gene Editor electroporator (BEX CO., LTD, Tokyo, Japan) [59]. Embryos were implanted into pseudo-pregnant recipients according to standard procedures. Gene editing of offspring was assessed using PCR, T7 Endonuclease I assay, and Sanger sequencing of the target region. Primers for *Pnldc1* E30A genotyping PCR are 5’-TGTACAGCTGCTTACCTCCT-3’ and 5’-AACAAAAACCAGCCCGCAG-3’. PCR products were digested with Hpy166II (R0616S, NEB).

For the generation of *Pnldc1* flox mice by CRISPR-Cas9 targeting, two synthetic single guide (sg) RNAs (Synthego Corp., Redwood City, CA, USA) were used to make RNPs which were introduced along with a homology directed repair (HDR) template into C57BL/6N zygotes via pronuclear microinjection. Protospacer (N)_20_ and PAM sequences corresponding to sgRNAs were 5’-GTACAGCTGCTTACCTCCTG-GGG-3’ for *Pnldc1* intron 1, and 5’-TGTCTGGTAGGGTCTAACTA-AGG-3’ for *Pnldc1* intron 2. The HDR template was a double-stranded DNA fragment containing two loxP sites flanking exon 2 of *Pnldc1* with the following sequence: 5’- CTGTTTTCCATGACTGTGTACAGCTGCTTACCTCGGATCCATAACTTCGTATAGCATACATTATACGAAGTTATCTGGGGCATTTGCAGTGTGAAAGGTGGTGCTTTGCTCTGTGCCTGAGGATTTTTGTCTGTTTTGTGCTCCCGTAGGTCTGGATATAGAGTTCACAGGTCTGCGTTCAAACTTGTCTCGGCCCCAACAGATCAGGTACTAATTACTGTCTTACCTCACCGGTGTCTCCTTAGATAACTTCGTATAGCATACATTATACGAAGTTATGAATTCTTAGACCCTACCAGACAAACATTGTGTCTGATCAG-3’. To generate *Pnldc1* cKO mice, *Stra8*-Cre transgenic mice (017490, Jackson Laboratory) were bred with *Pnldc1* flox/flox mice using the strategy described in S4B Fig. Primers for *Pnldc1* flox genotyping PCR are 5’-CCTGGGAACTGGTGTTTGGT-3’ and 5’-AGCCTATCAGCATTTGGCCA-3’. Primers for *Stra8*-Cre genotyping PCR are 5’-GTGCAAGCTGAACAACAGGA-3’ and 5’-AGGGACACAGCATTGGAGTC-3’. Primers for internal control in *Stra8*-Cre genotyping PCR are 5’-CTAGGCCACAGAATTGAAAGATCT-3’ and 5’-GTAGGTGGAAATTCTAGCATCATCC-3’ (S4C Fig).

*Pnldc1*^*-/-*^ and *Tdrkh*^*-/-*^ mice were generated and genotyped as previously described [40, 50].

### Plasmid construction

The full-length mouse *Pnldc1* and *Pnldc1*^*E30A*^ mutant cDNAs were amplified by PCR and cloned into pcDNA3-Flag (Flag tag at N-terminus) expression vector. The full-length mouse *Tdrkh* cDNA was amplified by PCR and cloned into pEGFP-N1 (GFP tag at C-terminus) expression vector.

### Histology

Mouse testes and epididymides were collected and fixed in Bouin’s solution (HT10132, Sigma-Aldrich) overnight at 4°C and embedded in paraffin. Histological sections were cut at 5 μm, dewaxed, rehydrated and stained with hematoxylin and eosin.

### Immunofluorescence

Mouse testes were fixed in 4% paraformaldehyde (PFA) in PBS overnight at 4°C and embedded in paraffin. Testis sections were cut at 5 μm, dewaxed and rehydrated. Antigen retrieval was performed in Tris-EDTA buffer (pH 9.0) or sodium citrate buffer (pH 6.0). Testis sections were blocked in 5% normal goat serum (NGS) at room temperature (RT) for 30 min. Testis sections were then incubated with anti-MIWI (1:100; 2079, Cell Signaling Technology), anti-MILI (1:100; PM044, MBL), anti-TDRKH (1:100; 13528-1-AP, Proteintech), anti-LINE1 ORF1 (1:800), anti-MIWI2 (1:50; ab21869, Abcam) or FITC-conjugated mouse anti-γH2AX (1:500; 16-202A, Millipore) in 5% NGS at 37°C for 2 h. After washing with PBS, sections were incubated with Alexa Fluor 555 goat anti-rabbit IgG (1:500; A21429, Thermo Fisher Scientific) at RT for 1 h and mounted using Vectashield mounting media with DAPI (H-1200, Vector Laboratories). Fluorescence was photographed using Fluoview FV1000 confocal microscope.

### Western blotting

Mouse testes were collected and homogenized in RIPA buffer (J63306-AP, Thermo Fisher Scientific) with protease inhibitor (A32965, Thermo Fisher Scientific). Protein lysates were separated by 4–20% polyacrylamide gels (4561096, Bio-Rad) and transferred to PVDF membranes. The membranes were blocked in 5% non-fat milk at RT for 30 min and subsequently incubated with primary antibodies in 5% non-fat milk overnight at 4°C. The primary antibodies used were anti-MIWI (1:1000; 2079, Cell Signaling Technology), anti-MILI (1:2000; PM044, MBL) anti-TDRKH (1:4000; 13528-1-AP, Proteintech), or HRP-conjugated mouse anti-β-actin (1:5000; A3854, Sigma-Aldrich). Membranes were washed with TBST and incubated with HRP-conjugated goat anti-rabbit IgG (1:5000; 1706515, Bio-Rad) at RT for 1 h followed by chemiluminescent detection with ECL Substrate (1705060, Bio-Rad).

### Transmission electron microscopy

Mouse testes were fixed in 2.5% glutaraldehyde in 0.1 M cacodylate buffer overnight at 4°C. After washing with 0.1 M cacodylate buffer, the testes were post-fixed in 1% osmium tetroxide in 0.1 M cacodylate buffer at RT for 2 h. The testes were then dehydrated in gradient series of acetone, infiltrated and embedded in Spurr’s resin. Ultrathin sections were cut at 70 nm and post-stained with uranyl acetate and lead citrate. Images were taken with JEOL 1400 Flash Transmission Electron Microscope (Japan Electron Optics Laboratory, Japan).

### Immunoprecipitation and mass spectrometry (IP-MS)

Mouse testes were collected and homogenized using lysis buffer (25 mM Tris-HCl pH = 7.4, 150 mM NaCl, 1% Triton X-100) with protease inhibitor (04693132001, Sigma-Aldrich) and PhosSTOP (4906845001, Sigma-Aldrich). The lysates were pre-cleared using Protein A agarose beads (11134515001, Sigma-Aldrich) at 4°C for 2 h. 10 μL anti-TDRKH (13528-1-AP, Proteintech) antibody was added to the lysates and incubated at 4°C overnight followed by Protein A agarose beads incubation at 4°C for 3 h. The beads were washed in lysis buffer 5 times and in PBS 5 times. Bound proteins were eluted with 2% SDS (w/v) for 10 min at 90°C. The eluted proteins were digested by trypsin (T1426, Sigma-Aldrich) according to the single-pot, solid-phase-enhanced sample-preparation protocol as described [60]. Peptides were resuspended in 20 μL sample buffer (0.1% formic acid and 2% acetonitrile in LC/MS grade water) and subjected to downstream RPLC-MS/MS analysis.

For RPLC-MS/MS, an EASY-RPLC 1200 system and an Orbitrap Exploris 480 mass spectrometer (Thermo Fisher Scientific) were used. 4 μL of the peptides was separated on a home-packed C18 separation column at a flow rate of 230 nL/min using an 8%-55%-100% 110-min gradient. Mobile phase A was 0.1% (v/v) formic acid in water, and mobile phase B was 80% (v/v) acetonitrile and 0.1% (v/v) formic acid in water. For the general parameters of mass spectrometer, the electrospray voltage was set to 2.2 kV, and the ion transfer tube temperature was 320°C. The MS/MS experiments were performed using data-dependent acquisition (DDA) and applied 10 scans for each cycle. The mass resolution was set to 120,000 (at m/z 200) for full MS scans and 60,000 (at m/z 200) for MS/MS scans.

For MS data analysis, all MS raw files were analyzed with Proteome Discoverer 2.2 (Thermo Fisher Scientific) by SEQUEST HT search engine. Peptides were searched against the UniProt mouse proteome database (UP000000589), and the false discovery rate (FDR%) was evaluated at 1% for PSM and peptide through target-decoy database search approach [61, 62].

### Immunoprecipitation of piRNAs and proteins

Mouse testes were collected and homogenized using lysis buffer (20 mM HEPES pH 7.3, 150 mM NaCl, 2.5 mM MgCl_2_, 0.2% NP-40, and 1 mM DTT) with protease inhibitor (A32965, Thermo Fisher Scientific) and RNase inhibitor (N2615, Promega). The lysates were pre-cleared using Protein A agarose beads (11134515001, Sigma-Aldrich) at 4°C for 2 h. Anti-MIWI (2079, Cell Signaling Technology) or anti-MILI (PM044, MBL) antibody together with Protein A agarose beads were added to the lysates and incubated at 4°C for 4 h. The beads were washed in lysis buffer 5 times. Immunoprecipitated RNAs were isolated from the beads using Trizol reagent (15596026, Thermo Fisher Scientific) for piRNA labeling or small RNA library construction. For protein detection, immunoprecipitated beads were boiled in protein loading buffer for 5 min. Western blotting of MIWI or MILI was performed as described above.

### Detection of piRNAs

Total RNA was extracted from mouse testes using Trizol reagent (15596026, Thermo Fisher Scientific). Total RNA or immunoprecipitated RNA (MIWI or MILI) was de-phosphorylated with Shrimp Alkaline Phosphatase (M0371, NEB) and end-labeled using T4 polynucleotide kinase (M0201, NEB) and [γ-^32^P] ATP (NEG002A250UC, PerkinElmer). The ^32^P-labeled RNA was separated by 15% Urea-PAGE gel. Radioactive signal was detected by exposing the gel on phosphorimager screen followed by scanning on the Typhoon scanner (GE Healthcare).

### Small RNA libraries and bioinformatics

Small RNA libraries from immunoprecipitated RNAs or total RNA were prepared using Small RNA Library Prep Kit (E7300, NEB) following the manufacturer’s instructions. Multiple libraries with different barcodes were pooled and sequenced with the Illumina HiSeq 4000 or NovaSeq 6000 platform (MSU Genomic Core Facility).

Sequenced reads were processed with fastx_clipper (http://hannonlab.cshl.edu/fastx_toolkit/index.html) to clip the sequencing adapter read-through. Clipped reads were filtered by length (24–48 nt) and aligned to the following sets of sequences: piRNA clusters, coding RNAs, non-coding RNAs, repeats, introns, and other. Alignments were performed with Bowtie (one base mismatch allowed). Repeats included classes of repeats as defined by RepeatMasker.

### Fixed cell immunofluorescence

HeLa cells were cultured in Chamber Slides (154534, Thermo Fisher Scientific) and transfected with indicated plasmids using Lipofectamine 2000 (11668019, Thermo Fisher Scientific). After 24 h, HeLa cells were fixed with 4% PFA for 15 min, incubated in 0.2% Triton X-100 in PBS for 20 min and blocked in 5% NGS at RT for 30 min. HeLa cells were then incubated with anti-FLAG antibody (1:100; F1804, Sigma-Aldrich) overnight at 4°C. After washing with PBS, HeLa cells were incubated with Alexa Fluor 555 goat anti-mouse IgG (1:200; A21422, Thermo Fisher Scientific) at RT for 1 h and mounted using Vectashield mounting media with DAPI (H-1200, Vector Laboratories). Fluorescence was photographed using Fluoview FV1000 confocal microscope.

### Trimming assay

HEK293T cells were transfected with indicated plasmids using Lipofectamine 2000 (11668019, Thermo Fisher Scientific). After 24 h, cells were lysed in trimming buffer (20 mM HEPES-KOH pH 7.0, 100 mM NaCl, 1.5 mM MgCl_2_, 0.1 mM EDTA, 0.1 mM DTT, protease inhibitor and RNase inhibitor) containing 1% Triton X-100 and immunoprecipitation was performed using anti-FLAG M2 agarose beads (A2220, Sigma-Aldrich). After immunoprecipitation, the beads were resuspended in 20 μl trimming buffer containing 0.5 uM 5’FAM labeled RNA oligo (N9A15: UGCCGCCCCAAAAAAAAAAAAAAA, or UCAG6: UCAGUCAGUCAGUCAGUCAGUCAG) and incubated in the shaker at 37°C for 1 h. Samples were then separated by 20% Urea-PAGE gel at 250 V for 1 h and the fluorescent signal was captured by the ChemiDoc System (Bio-Rad). For protein detection, immunoprecipitated beads were boiled in protein loading buffer for 5 min. Western blotting was performed as described above using anti-FLAG antibody (1:1000; F1804, Sigma-Aldrich) or anti-GFP antibody (1:10000; Ab290, Abcam) and secondary antibodies HRP-conjugated goat anti-mouse IgG (1:5000; 1706516, Bio-Rad) or goat anti-rabbit IgG (1:5000; 1706515, Bio-Rad).

### Statistical analysis

All data are presented as mean ± SEM and all statistical analyses between groups were performed by unpaired t-test.

## Supporting information

S1 Fig Targeting of the *Pndlc1* locus to generate E30A mutant mice. **(A)** The location of gRNA target protospacer, PAM, and the double stranded break following Cas9 cleavage are indicated on the WT allele. Modified codon E30A (GAG > Gct) is highlighted. The resulting edited allele sequence and translation are presented. **(B)** *Pnldc1* WT and E30A mutation are shown by Sanger DNA sequencing.
(TIF)

S2 Fig Expression and localization of MIWI, MILI, and TDRKH in *Pnldc1*^*E30A/-*^ testes. **(A)** Western blotting of MIWI, MILI, and TDRKH in adult *Pnldc1*^*+/-*^, *Pnldc1*^*E30A/-*^, and *Pnldc1*^*-/-*^ testes. β-actin served as loading control. **(B)** Co-immunostaining of MIWI, MILI, or TDRKH (red) with γH2AX (green) in adult *Pnldc1*^*+/-*^, *Pnldc1*^*E30A/-*^, and *Pnldc1*^*-/-*^ spermatocytes. DNA was stained with DAPI. Protein aggregation is indicated by arrows. Scale bar, 10μm. **(C)** Transmission electron microscopy of round spermatids from adult *Pnldc1*^*+/-*^, *Pnldc1*^*E30A/-*^, and *Pnldc1*^*-/-*^ testes. Chromatoid bodies are indicated by arrows. Scale bar, 5μm. Results shown in (A)-(C) are representative of 3 biological replicates.
(TIF)

S3 Fig Extended piRNA 3’ ends in adult *Pnldc1*^*E30A/-*^ testis. **(A)** Nucleotide distributions at the first position in total piRNA, MIWI-piRNAs, and MILI-piRNAs from adult *Pnldc1*^*+/-*^, *Pnldc1*^*E30A/-*^, and *Pnldc1*^*-/-*^ testes. **(B)** Two examples of read alignments between MILI-piRNAs and piRNA clusters from adult *Pnldc1*^*+/-*^, *Pnldc1*^*E30A/-*^, and *Pnldc1*^*-/-*^ testes. The genomic locations of the two piRNA clusters are shown at the bottom.
(TIF)

S4 Fig Targeting of the *Pndlc1* locus to generate *Pnldc1* flox mice. **(A)** The location of gRNAs and the double stranded break following Cas9 cleavage are indicated on the WT allele. The HDR template containing two loxP sequences is indicated on the edited allele. **(B)** The breeding strategy to generate *Stra8*-Cre^+^; *Pnldc1* flox/- (*Pnldc1* cKO) mice. **(C)** Genotyping PCR of *Pnldc1* flox allele and *Stra8*-Cre allele. An internal control PCR was used to indicate the presence of genomic DNA.
(TIF)

S5 Fig Expression and localization of MIWI, MILI, and TDRKH in *Pnldc1* cKO spermatocytes. **(A)** Western blotting of MIWI, MILI, and TDRKH in adult control and *Pnldc1* cKO testes. β-actin served as loading control. **(B)** Co-immunostaining of MIWI, MILI, or TDRKH (red) with γH2AX (green) in adult control and *Pnldc1* cKO spermatocytes. DNA was stained with DAPI. Protein aggregation is indicated by arrows. Scale bar, 10μm. **(C)** Transmission electron microscopy of round spermatids from adult control and *Pnldc1* cKO testes. Chromatoid bodies are indicated by arrows. Scale bar, 5μm. **(D)** Co-immunostaining of MIWI (red) or LINE1 ORF1 (red) with γH2AX (green) in P18 control, *Pnldc1* cKO, *Pnldc1*^*-/-*^, and *Pnldc1*^*E30A/-*^ spermatocytes. DNA was stained with DAPI. Scale bar, 20μm. Results shown in (A)-(D) are representative of 3 biological replicates.
(TIF)

S1 Table Abbreviated table showing PNLDC1-TDRKH interaction in IP-MS. (DOCX)

S2 Table TDRKH IP-MS list for *Pnldc1* control testes. (XLSX)

S3 Table TDRKH IP-MS list for *Pnldc1* E30A testes. (XLSX)

S4 Table Raw data for figures. (XLSX)
