# Supplementary figures and images for: PNLDC1 catalysis and postnatal germline function are required for piRNA trimming, LINE1 silencing, and spermatogenesis in mice
Source: PLoS Genet. 2024 Sep 23;20(9):e1011429. doi: 10.1371/journal.pgen.1011429 (PMC11449332; doi:10.1371/journal.pgen.1011429)

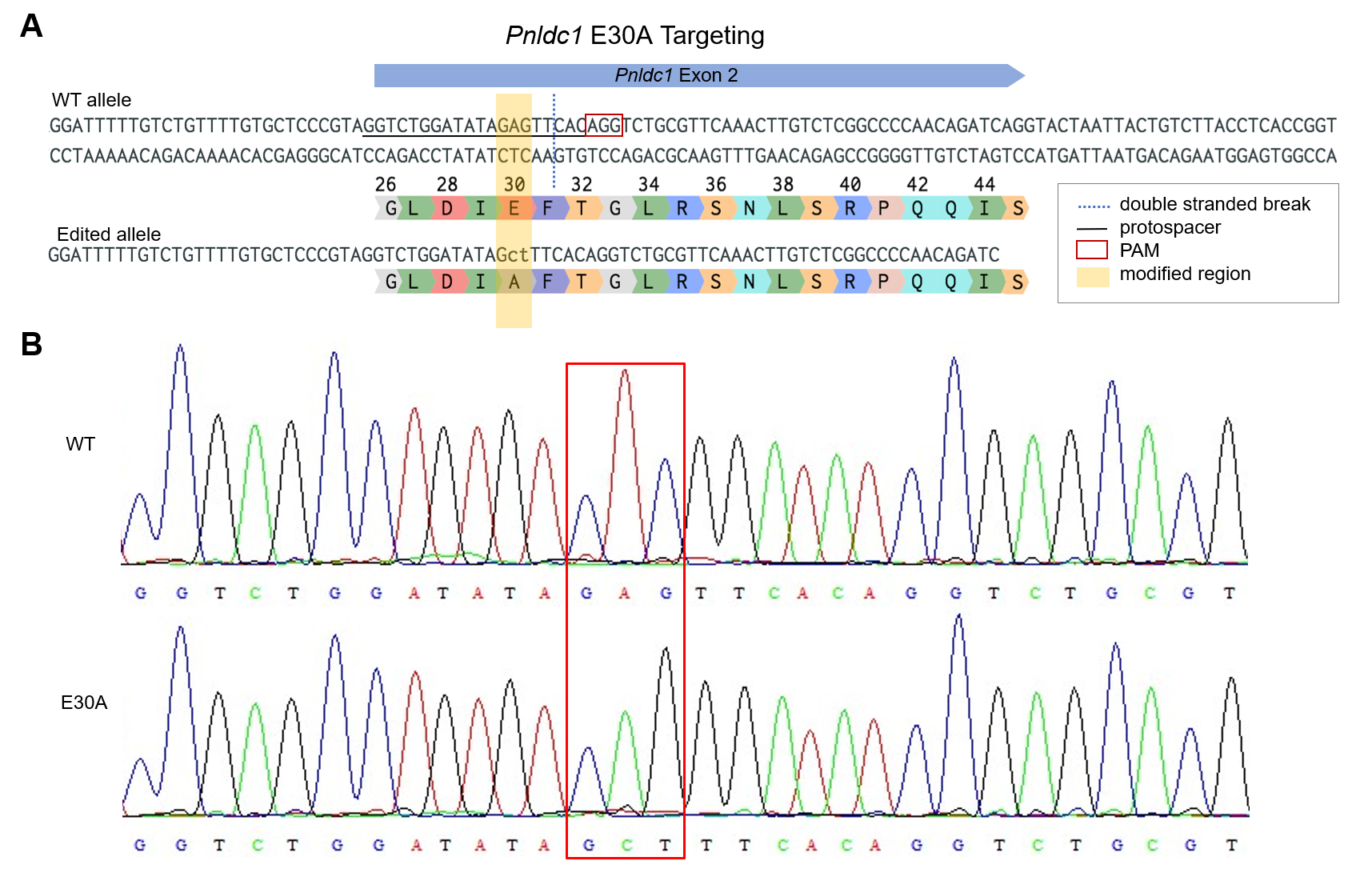

Supplement: S1 Fig — (A) The location of gRNA target protospacer, PAM, and the double stranded break following Cas9 cleavage are indicated on the WT allele. Modified codon E30A (GAG > Gct) is highlighted. The resulting edited allele sequence and translation are presented. (B) Pnldc1 WT and E30A mutation are shown by Sanger DNA sequencing. (TIF) [file pgen.1011429.s001.tif]

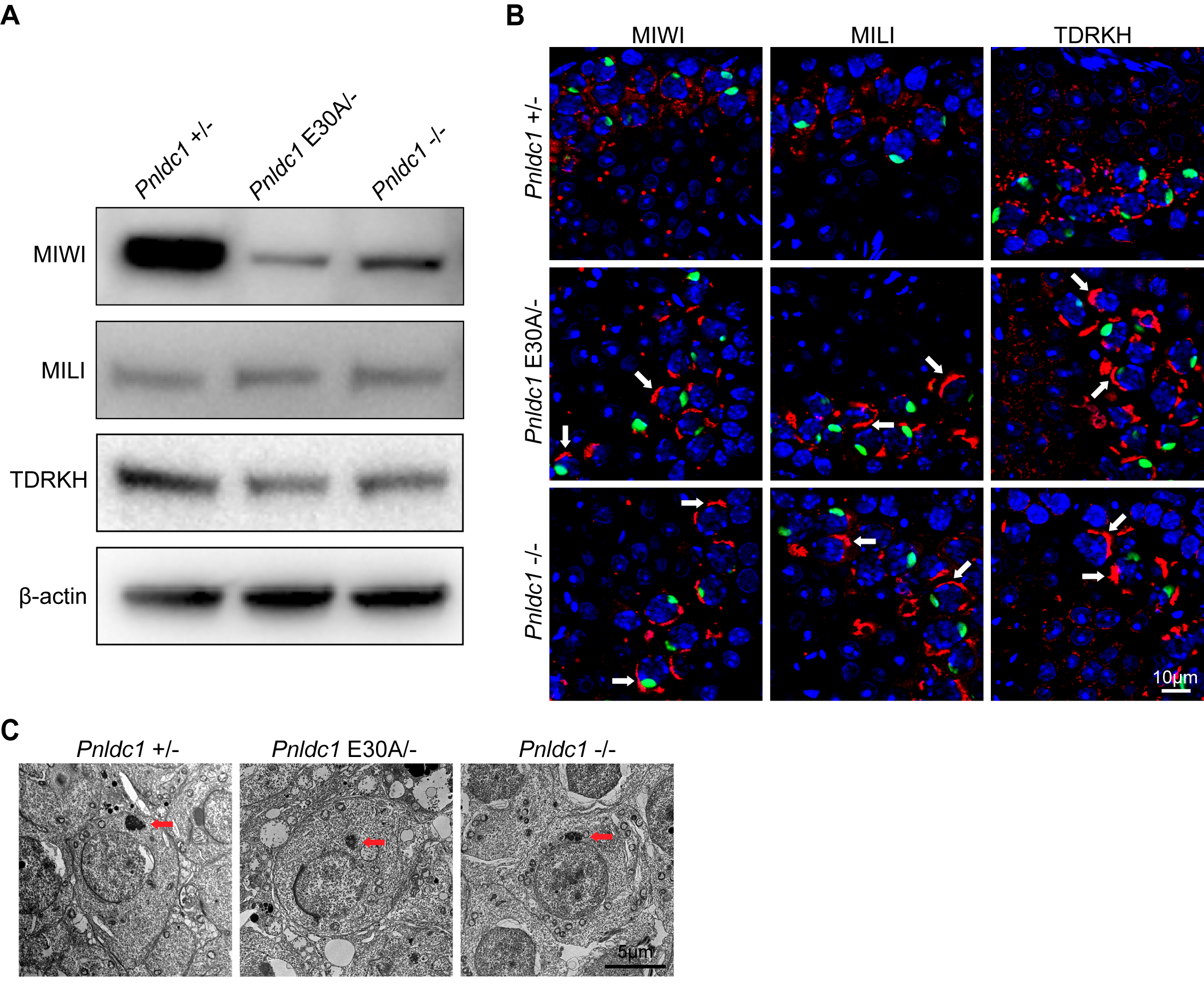

Supplement: S2 Fig — (A) Western blotting of MIWI, MILI, and TDRKH in adult Pnldc1+/-, Pnldc1E30A/-, and Pnldc1-/- testes. β-actin served as loading control. (B) Co-immunostaining of MIWI, MILI, or TDRKH (red) with γH2AX (green) in adult Pnldc1+/-, Pnldc1E30A/-, and Pnldc1-/- spermatocytes. DNA was stained with DAPI. Protein aggregation is indicated by arrows. Scale bar, 10μm. (C) Transmission electron microscopy of round spermatids from adult Pnldc1+/-, Pnldc1E30A/-, and Pnldc1-/- testes. Chromatoid bodies are indicated by arrows. Scale bar, 5μm. Results shown in (A)-(C) are representative of 3 biological replicates. (TIF) [file pgen.1011429.s002.tif]

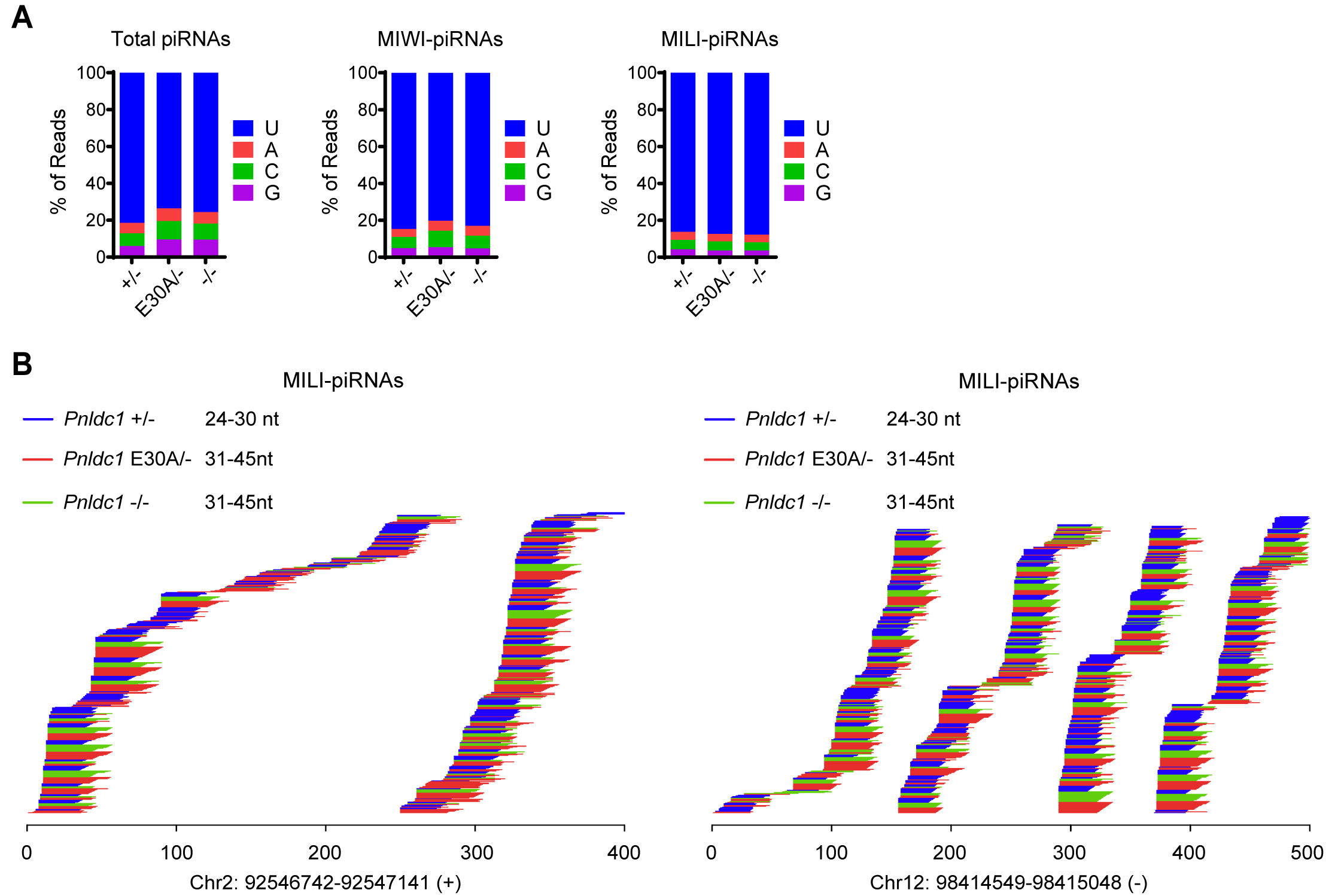

Supplement: S3 Fig — (A) Nucleotide distributions at the first position in total piRNA, MIWI-piRNAs, and MILI-piRNAs from adult Pnldc1+/-, Pnldc1E30A/-, and Pnldc1-/- testes. (B) Two examples of read alignments between MILI-piRNAs and piRNA clusters from adult Pnldc1+/-, Pnldc1E30A/-, and Pnldc1-/- testes. The genomic locations of the two piRNA clusters are shown at the bottom. (TIF) [file pgen.1011429.s003.tif]

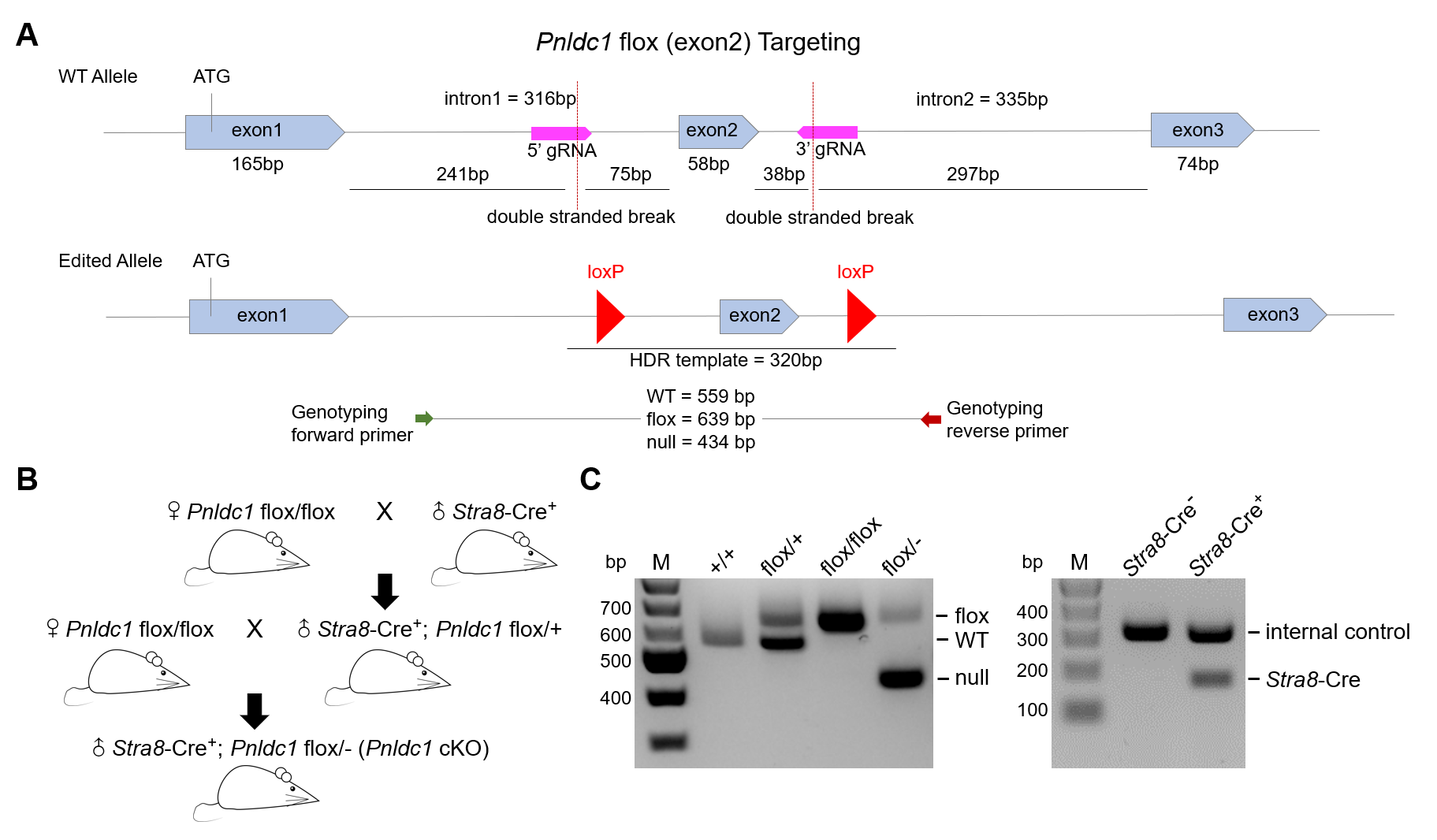

Supplement: S4 Fig — (A) The location of gRNAs and the double stranded break following Cas9 cleavage are indicated on the WT allele. The HDR template containing two loxP sequences is indicated on the edited allele. (B) The breeding strategy to generate Stra8-Cre+; Pnldc1 flox/- (Pnldc1 cKO) mice. (C) Genotyping PCR of Pnldc1 flox allele and Stra8-Cre allele. An internal control PCR was used to indicate the presence of genomic DNA. (TIF) [file pgen.1011429.s004.tif]

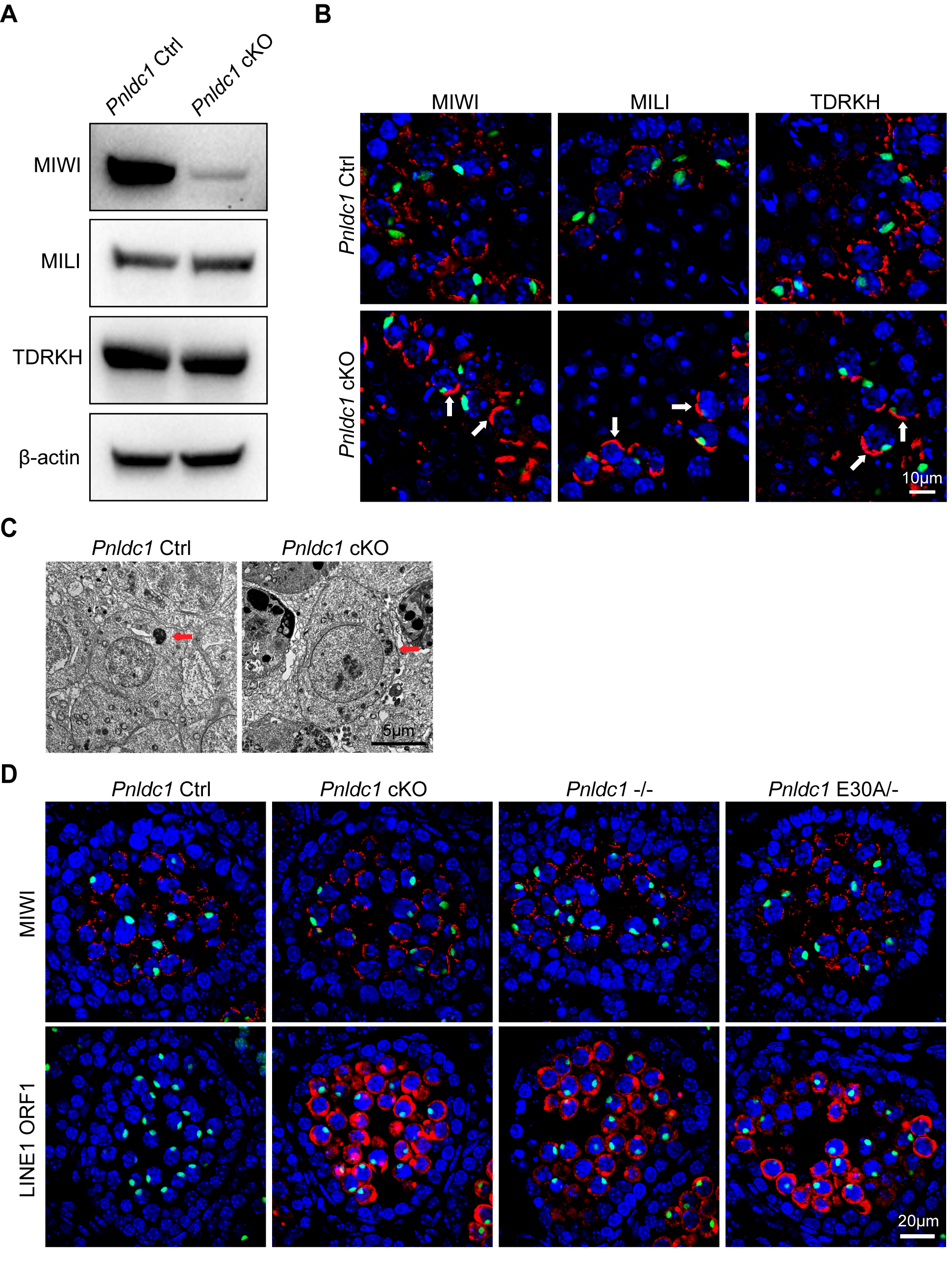

Supplement: S5 Fig — (A) Western blotting of MIWI, MILI, and TDRKH in adult control and Pnldc1 cKO testes. β-actin served as loading control. (B) Co-immunostaining of MIWI, MILI, or TDRKH (red) with γH2AX (green) in adult control and Pnldc1 cKO spermatocytes. DNA was stained with DAPI. Protein aggregation is indicated by arrows. Scale bar, 10μm. (C) Transmission electron microscopy of round spermatids from adult control and Pnldc1 cKO testes. Chromatoid bodies are indicated by arrows. Scale bar, 5μm. (D) Co-immunostaining of MIWI (red) or LINE1 ORF1 (red) with γH2AX (green) in P18 control, Pnldc1 cKO, Pnldc1-/-, and Pnldc1E30A/- spermatocytes. DNA was stained with DAPI. Scale bar, 20μm. Results shown in (A)-(D) are representative of 3 biological replicates. (TIF) [file pgen.1011429.s005.tif]
