# Supplementary material for: PNLDC1 catalysis and postnatal germline function are required for piRNA trimming, LINE1 silencing, and spermatogenesis in mice
Source: PLoS Genet. 2024 Sep 23;20(9):e1011429. doi: 10.1371/journal.pgen.1011429 (PMC11449332; doi:10.1371/journal.pgen.1011429)
Supplement: S1 Table — (DOCX) [file pgen.1011429.s006.docx]

**Table S1: abbreviated table showing PNLDC1-TDRKH interaction in IP-MS**

TDRKH IP-MS in *Pnldc1^+/-^* testes

| Accession | Description | Sum PEP Score | Coverage [%] | # Peptides |
| --- | --- | --- | --- | --- |
| Q80VL1 | Tudor and KH domain-containing protein OS=Mus musculus OX=10090 GN=Tdrkh PE=1 SV=1 | 182.022 | 48 | 30 |
| B2RXZ1 | Poly(A)-specific ribonuclease PNLDC1 OS=Mus musculus OX=10090 GN=Pnldc1 PE=1 SV=1 | 10.508 | 9 | 4 |

TDRKH IP-MS in *Pnldc1^E30A/-^* testes

| Accession | Description | Sum PEP Score | Coverage [%] | # Peptides |
| --- | --- | --- | --- | --- |
| Q80VL1 | Tudor and KH domain-containing protein OS=Mus musculus OX=10090 GN=Tdrkh PE=1 SV=1 | 246.463 | 58 | 34 |
| B2RXZ1 | Poly(A)-specific ribonuclease PNLDC1 OS=Mus musculus OX=10090 GN=Pnldc1 PE=1 SV=1 | 36.352 | 11 | 6 |
